# A Case of Accelerated Rehabilitation: Immediate Loading of Full Mouth Basal Implants

**DOI:** 10.7759/cureus.65556

**Published:** 2024-07-28

**Authors:** Nikhar Wadhwani, Nitin Bhola, Bhushan P Mundada, Arushi Beri, Vaishnavi Patekar

**Affiliations:** 1 Oral and Maxillofacial Surgery, Datta Meghe Institute of Higher Education and Research, Nagpur, IND; 2 Oral and Maxillofacial Surgery, Sharad Pawar Dental College and Hospital, Datta Meghe Institute of Higher Education and Research, Wardha, IND; 3 Prosthodontics, Sharad Pawar Dental College and Hospital, Datta Meghe Institute of Higher Education and Research, Wardha, IND; 4 Dentistry, Sharad Pawar Dental College and Hospital, Datta Meghe Institute of Higher Education and Research, Wardha, IND

**Keywords:** pterygoid implants, zygomatic implants, basal implants, full mouth rehabilitation, implantology, dental implants, implants

## Abstract

Basal implantology, also known as bi-cortical implantology, is an advanced system using the dense basal cortical bone for anchoring dental implants, ideal for patients with severe bone loss. Unlike traditional implants that require healthy cancellous bone, basal implants engage with the cortical bone, providing superior stability and durability. These implants can be immediately loaded due to their load-bearing capacity, making them a quick and effective solution for full mouth rehabilitation. A case report of a 55-year-old male with significant bone resorption illustrates the benefits of basal implants. Conventional endosteal implants were not viable, so nine basal implants in the mandible and a combination of basal, pterygoid, and zygomatic implants in the maxilla were used. Within 72 hours, the patient experienced significant improvements in chewing, aesthetics, and overall oral function. Basal implants offer a reliable alternative for patients with challenging anatomical conditions, demonstrating excellent functional and aesthetic outcomes.

## Introduction

Dental implants are a modern solution for replacing missing teeth, consisting of titanium posts surgically inserted into the jawbone. These posts fuse with the bone, providing a stable foundation for prosthetic teeth. Dental implants offer a natural look and feel, improving chewing efficiency and speech. They also preserve jawbone structure and can last a lifetime with proper care, making them a reliable, long-term solution for restoring smiles [1].

Problems related to oral health like severe occlusal wear, substantial bone resorption, advanced periodontal disease, and extensive tooth loss require full mouth rehabilitation. Although this is usually done by endosteal implants, patients with severe bone loss are not ideal cases for them as they require healthy cancellous bone for retention.

Basal implantology, also known as bi-cortical implantology, is an advanced dental implant system that utilizes the dense basal cortical portion of the jaw bones for anchoring implants. Unlike traditional implants that rely on cancellous bone, basal implants are designed to engage with the strong cortical bone, which offers superior stability and durability. This type of bone is less susceptible to resorption, making basal implants a reliable long-term solution for tooth replacement, especially beneficial for patients with significant bone loss [2].

These implants were specifically designed for use in jawbones affected by atrophy, engaging with the infection and resorption-resistant basal bone, and are capable of immediate loading due to their load-bearing capacity [3]. The masticatory forces are directly transferred to the cortical bone.

We describe the report of a case in which, due to severe bone resorption, endosteal implants could not be placed; hence, we opted for rehabilitation with basal implants. After placement, they were immediately loaded, and within 72 hours, our patient had remarkable improvements: he was able to chew comfortably, with a significantly enhanced smile and restored vertical facial height, and their overall oral function was fully rehabilitated.

## Case presentation

**Figure 1:**
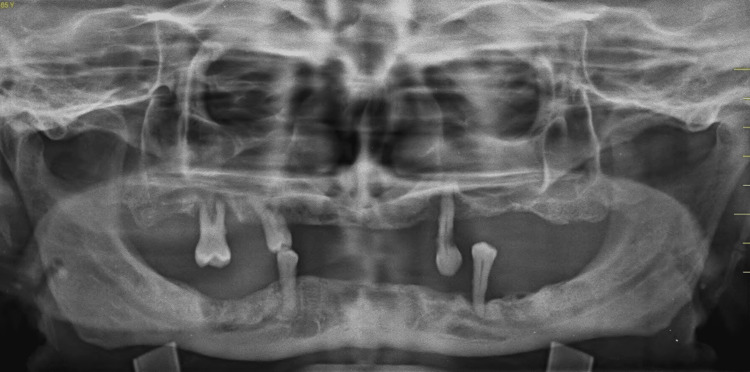
Orthopantomogram of the patient showing resorbed ridges and insufficient bone height

A 55-year-old male patient reported to our facility with the chief complaint of missing teeth in the lower right and left back regions of his jaws and wanted to get his missing teeth replaced. The patient gave a history of exfoliation of the lower anterior teeth two months ago. On intraoral examination, it was observed that all the remaining teeth were periodontally compromised (Grade III mobility was elicited). An orthopantomogram (OPG) was done, revealing severely resorbed ridges and insufficient bone height (Figure 1). 

**Figure 2:**
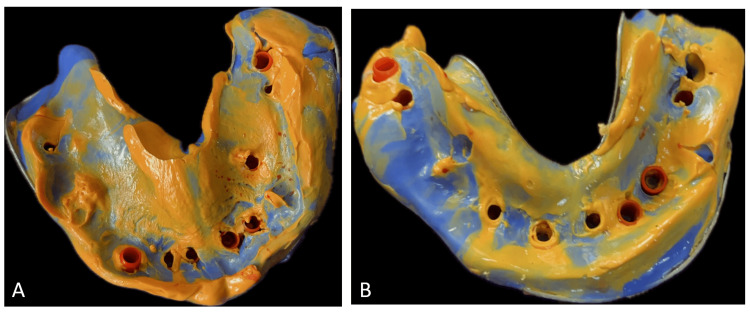
Putty light body impression of (A) maxillary arch and (B) mandibular arch

A cone-beam computed tomography (CBCT) evaluation was done, which revealed insufficient bone for conventional endosteal implants. Additionally, the bone type was classified as D3-D4. Therefore, a treatment option involving basal implants was discussed in detail with the patient, and nine basal implants in the mandible, along with four basal, three pterygoid, and one zygomatic basal implant in the maxilla, were planned for the patient and were immediately loaded within 72 hours. After the placement of implants, the impression was made with putty and light body impression material (Figure 2).

**Figure 3:**
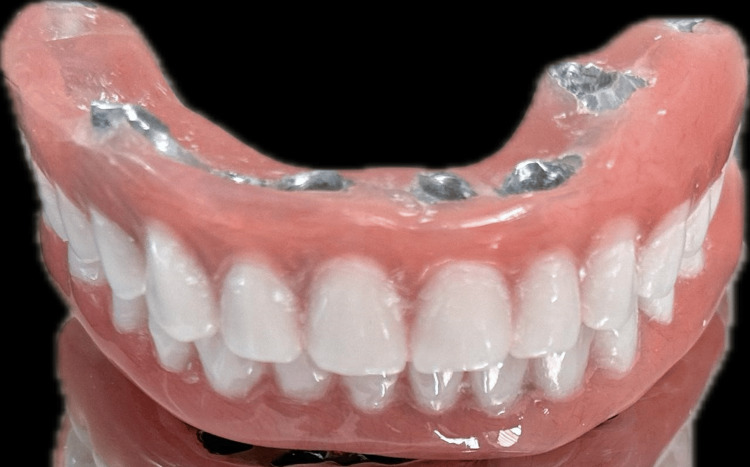
Porcelain fused to metal prosthesis after cementation

A jaw relation was made, and a fixed prosthesis 3 (FP3) was planned, which was followed by metal try-in and layering with acrylic restoration and cementation of the final prosthesis (Figure 3).

**Figure 4:**
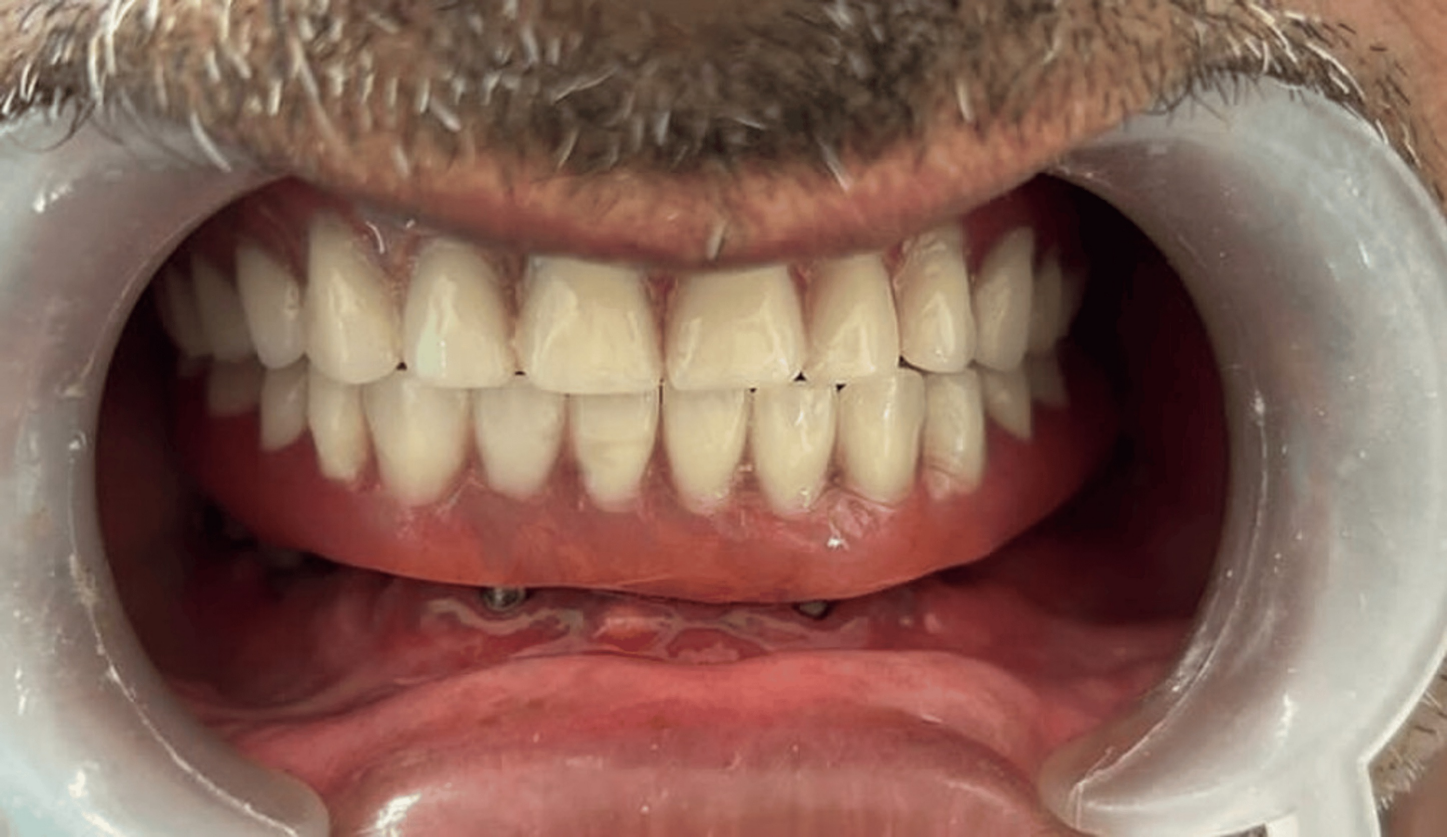
Postoperative frontal view of the patient

**Figure 5:**
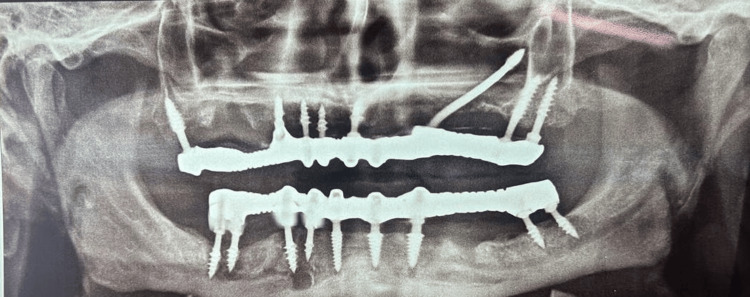
Postoperative OPG of the patient OPG: Orthopantomogram

The implant-protected occlusal schemes were selected, and any occlusal interferences in centric, protrusive, and lateral movements were removed. Figure 4 shows the postoperative frontal view of the patient, and Figure 5 shows the postoperative OPG.

## Discussion

Dental implants offer numerous advantages over traditional removable and fixed tooth-supported prostheses, making them the most widely accepted treatment option for replacing lost teeth. However, the conventional method of placing endosteal implants has several drawbacks. The most common issues include the design of multiunit implants with multiple interconnecting screws, delayed loading, and the necessity for adequate bone volume and density. Consequently, the basic dental implant design, initially created by Dr. Gérard Scortecci in 1980, has undergone multiple modifications to evolve into a single-piece implant [4,5].

In the traditional Brånemark approach to implant insertion, the alveolar bone of the jaws is utilized. This bone is more likely to resorb and disappear as teeth are extracted or function declines. In contrast, basal implants are placed within the basal bone, which has several benefits. Unlike the alveolar bone, the basal bone is less likely to resorb, provides strong support for the implants, and has limited vascularity, reducing the risk of infections. Biomechanically, basal implants anchor on the dense basal bone, offering exceptional primary stability. Patients benefit from fast function restoration and a shorter recovery period. The flapless technique, a less invasive treatment option, is ideal for individuals with significant bone loss, where conventional implants would fail without considerable augmentation. This technique eliminates surgical damage, speeds up healing, and reduces expenses and time needed, providing a more cautious approach. [6-8]

Basal implants can be loaded immediately after placement due to their ability to achieve excellent primary stability in dense cortical bone, making them more reliable than before. To ensure stability, restrictive splinting of the metal framework should be done as soon as possible, as bone remodeling begins within 72 hours and can weaken the peri-implant bone structures. Splinting helps distribute the masticatory forces generated in the bone surrounding the implants to other cortical areas [9,10].

In this case, nine implants were inserted into the mandible, and four basal, three pterygoid, and one zygomatic implants were placed in the maxilla due to insufficient bone height, making basal implant treatment necessary. The use of basal implants demonstrated significant benefits for individuals with challenging anatomical and clinical situations. The unique architecture of basal implants allowed for a rapid loading strategy, which shortened treatment times and required fewer invasive procedures. During the follow-up period, the patient exhibited good functional and aesthetic outcomes with no reported issues. These findings underscore the potential of basal implants as a viable and practical solution for poor bone health, offering an effective alternative to conventional implant techniques.

## Conclusions

A new and reliable therapy option for those who need to quickly restore resorbed ridges is basal implantology. The basal implant treatment is now accessible, safe, and affordable for all patients, including those with severe periodontitis and managed diabetes. Flapless techniques reduce bleeding, expedite recuperation times, and enhance patient outcomes even further. Based on the latest advancements in implant dentistry, which prioritize prosthetic-driven systems and rapid loading methods, these positive outcomes suggest that basal implants play a vital role in modern implantology and can greatly improve patients' quality of life. They also eliminate most disadvantages of traditional implantology.
